# Evaluation of Thermally Induced Degradation of Branched Polypropylene by Using Rheology and Different Constitutive Equations

**DOI:** 10.3390/polym8090317

**Published:** 2016-08-24

**Authors:** Jiri Drabek, Martin Zatloukal

**Affiliations:** Polymer Centre, Faculty of Technology, Tomas Bata University in Zlin, Vavreckova 275, 760 01 Zlin, Czech Republic; drabek@ft.utb.cz

**Keywords:** branched polypropylene, thermal degradation, uniaxial extensional viscosity, polymer melts, constitutive equations

## Abstract

In this work, virgin as well as thermally degraded branched polypropylenes were investigated by using rotational and Sentmanat extensional rheometers, gel permeation chromatography and different constitutive equations. Based on the obtained experimental data and theoretical analysis, it has been found that even if both chain scission and branching takes place during thermal degradation of the tested polypropylene, the melt strength (quantified via the level of extensional strain hardening) can increase at short degradation times. It was found that constitutive equations such as Generalized Newtonian law, modified White-Metzner model, Yao and Extended Yao models have the capability to describe and interpret the measured steady-state rheological data of the virgin as well as thermally degraded branched polypropylenes. Specific attention has been paid to understanding molecular changes during thermal degradation of branched polypropylene by using physical parameters of utilized constitutive equations.

## 1. Introduction

Polyolefins are one of the most globally produced and widely used polymeric materials in the marketplace. The most widespread representative of polyolefins is polypropylene (PP) for its higher melting point, lower density, chemical resistance, rigidity, impact strength and low cost. For these properties, the PP can be used in many manufacturing processes such as compression, foaming, thermoforming, blow moulding, injection moulding, rotational moulding and extrusion coating. PP occurs in two basic types (linear, branched) and is usually processed in a melt state. The key problem with processing of conventional linear PP is its low melt strength, which may limit processing window considerably. Thus, it is not surprising that considerable progress has been made to enhance PP melt strength by introduction of long chain branching via different methods such as electron beam radiation [1,2,3,4,5,6], gamma radiation [1,7,8], UV radiation [9,10,11,12,13,14,15,16] or utilizing peroxides (usually in presence of multi-functional monomers) [17,18,19,20,21,22,23]. It has been reported that branched PPs have a predominantly star-like structure [5,24,25,26,27,28], which causes significant extensional strain hardening inducing a so-called “self-healing” effect leading to improved quality of products (for example, wall thickness uniformity or a better foam structure) due to homogeneous deformation in elongational flows [28,29,30,31,32], a more pronounced shear thinning behaviour, and a higher elasticity compared to linear polypropylene [19,23,33,34]. It is important to mention that the “topological stiffness” is typical for so-called semiflexible polymers such as comb, brush, star, H-shaped and multiarm (pom-pom) branched polymers (including highly branched dendronized polymers having a linear backbone with a very high number of attached dendritic side groups as the extreme case) [35,36,37,38,39,40,41,42,43,44,45], i.e., polymers, whose behaviour lies between perfectly flexible and perfectly rigid polymer chains and their molecular elasticity has impact on both single-chain and collective behaviour [46].

During processing of polypropylenes, degradation reactions can start to occur, which can significantly influence their molecular structure, narrow the processing window, as well as reduce basic properties of the final product considerably. The most attention has been focused on conventional linear PPs. In more detail, it has been shown that free-radical-induced degradation of PP, induced by different amounts of peroxide, leads to reduction in average molecular weight, polydispersity and melt viscosity [47,48,49,50,51,52,53]. Thermo-mechanical and/or thermo-oxidative degradation of PPs was investigated by using the multiple extrusion process [54,55,56], torque rheometer [57], rheometric mechanical spectrometer [58], and windy oven [59]. It has been demonstrated that Newtonian viscosity [55], recoverable shear compliance [55], molecular weight [54], loss and storage moduli [58] monotonically decrease with the degradation time mainly due to chain scission of macromolecules driven via β-scission reaction, breakdown of peroxy radicals and shear [54]. It has been found that during thermo-mechanical degradation, the chain scission at low molecular weight (*M*_W_) is random (i.e., *M*_W_-independent) but it is increasing with *M*_W_ at higher *M*_W_, which is in good agreement with mechanical degradation theory of Bueche, which states that “the probability of chain scission is higher for the high *M*_W_ chains to yield smaller molecules of about half the original size” [56]. The situation was found to be even more complex during thermo-oxidative degradation, during which chain scission can be kinetically favoured near the oxygen-centred radicals leading to a heterogeneous type of degradation and causing change from unimodal to bimodal MWD [59].

It has been shown that rheology can be used as a powerful tool for understanding degradation of different polymers due to its sensitivity to very small changes in the molecular structure of polymers, as well as for understanding their molecular structure via utilization of advanced constitutive equations, which are based on the molecular arguments [60,61,62,63,64,65]. In more detail, multimode eXtended Pom-Pom (XPP) and PTT-XPP models have been used to understand structural changes of PPs during peroxide-induced modification by the reactive extrusion process [60,65]. Even if these models have been found to be useful for a branching level quantification, there are some disadvantages connected with their usage. Firstly, model parameters’ identification process is rather complicated due to high mathematical complexity and huge number of adjustable parameters, which usually require their manual adjustment. Secondly, both models are based on the assumption that branched macromolecules take the “H” shape, which can result in poor model predictions, especially for PPs having different, e.g., star-like, structures [60].

Even if the degradation behaviour of conventional linear PP is well documented in the open literature, only a little or no information is known about structural changes during thermal degradation of branched polypropylenes, especially with respect to branching, which could be used for process stabilization and optimization of final product properties. With the aim to extend the knowledge in this area, thermal stability of branched polypropylene was investigated in this work by using shear and extensional rheology as well as the GPC technique. Specific attention was paid to quantification and interpretation of the measured data by using four different constitutive equations allowing manual-adjustment-free determination of their parameters as well as quantification/characterization of molecular changes during thermal degradation of branched PP.

## 2. Experimental

In this work, branched PP Daploy WB180HMS (Borealis Polyolefine, Linz, Austria) has been used. In the first step, the polymer was thermally degraded. For such a purpose, polymer was added into a Rosand RH7-2 twin bore capillary rheometer (Rosand Precision, Stourbridge, England), melted and kept at given times (1, 3, 5 and 7 h) at 240 °C. Obtained thermally degraded polymers together with the virgin polymer were consequently pressed to plates from which the final testing samples were prepared. Linear viscoelastic properties were measured on Advanced Rheometric Expansion System (ARES 2000 model, Rheometric Scientific, Piscataway, NJ, USA) at 170 °C, 180 °C and 190 °C in parallel plates mode. In order to capture Newtonian plateau, shear creep measurements were performed at the reference temperature equal to 180 °C. Transient uniaxial extensional viscosity, η_E_, was determined at 170 °C, 180 °C and 190 °C on Sentmanat Extensional Rheometer (SER-HV-A01 model, Xpansion Instruments, Tallmadge, OH, USA [66,67,68]) attached to ARES 2000 rotational rheometer and the obtained measured data were shifted to the reference temperature. It is important to mentioned that time-resolved mechanical spectroscopy was used to ensure that there is virtually no melt degradation during rheological characterization of tested polymer samples for the given temperature range (170 °C–190 °C). Finally, high temperature Gel Permeation Chromatography (GPC) was used to determine number average molecular weight, *M*_n_, weight average molecular weight, *M*_w_, Z-average molecular weight, *M*_z_, and *Z*+1-average molecular weight, *M*_z+1_, for all tested samples.

## 3. Theoretical

### 3.1. Generalized Newtonian Fluid Model

In this work, the recently proposed generalized Newtonian fluid model has been utilized [69,70,71]. Mathematically, it relates the extra stress tensor, τ, and deformation rate tensor, *D*, as

(1) $$\tau =2A^{1-\text{f}\left(I_{\left|D\right|},{\text{II}}_{D},{\text{III}}_{D}\right)}\eta {\left(II_{D}\right)}^{f\left(I_{\left|D\right|},{\text{II}}_{D},{\text{III}}_{D}\right)}D$$

where η(*II*_D_) represents well-known Carreau-Yasuda model (Equation (2)) and *f*(*I*_|D|_, *II*_D_, *III*_D_) is given by Equation (3).

(2) $$\eta \left(II_{D}\right)=\frac{\eta_{0}}{{\left[1+{\left(K_{1}\sqrt{II_{D}}\right)}^{a}\right]}^{\left(\frac{1-\text{n}}{a}\right)}}$$

(3) $$f\left(I_{\left|D\right|},II_{D},III_{D}\right)={\left\{\tanh\left[\alpha {\left(1+\frac{1}{4{\left(\sqrt{3}\right)}^{\;3}}\right)}^{-\psi }{\left(\left|1+\frac{III_{D}}{II_{D}^{3/2}}\right|\right)}^{\psi }\frac{\sqrt[{3}]{4\left|III_{D}\right|}+I_{\left|D\right|}}{3}+\beta \right]\frac{1}{\tanh(\beta )}\right\}}^{\zeta }$$

In this model, the viscosity is allowed to vary with the first invariant of the absolute value of deformation rate tensor *I*_|D|_ = *tr*(|D|), (where |D| is defined as the $\sqrt{D\cdot D}$) as well as on the second *II*_D_ = 2*tr*(D^2^), and third, *III*_D_ = det(D), invariants of D. The model utilizes four parameters (η_0_, *K*_1_, *a*, *n*) identifiable from shear viscosity, four parameters (*A*, α, β, ζ) identifiable from uniaxial extensional viscosity and one parameter (ψ), which can be determined from planar extensional viscosity. For the pure shear flow, the function *f*(*I*_|D|_, *II*_D_, *III*_D_) becomes equal to 1 and thus, the viscosity becomes a function of second invariant of deformation rate tensor *D* ($II_{D}={\dot{\gamma }}^{2}$) only. In the pure uniaxial extensional flow, where $I_{\left|D\right|}=\;2\dot{\varepsilon }$, $II_{D}=3{\dot{\varepsilon }}^{2}$, and $III_{D}={\dot{\varepsilon }}^{3}/4$, the function *f*(*I*_|D|_, *II*_D_, *III*_D_) becomes nonzero and model becomes capable of representing steady-state extensional flows for different polymer melts [69,70,71,72,73,74].

### 3.2. Modified White-Metzner Model

Modified White-Metzner constitutive equation is a simple Maxwell model for which the viscosity and relaxation time are allowed to vary with the second invariant of the deformation rate tensor [75]. It takes the following form:

(4) $$\tau +\frac{\lambda_{0}}{1+K_{2}II_{D}}\overset{\nabla }{\tau }=2\frac{\eta_{0}}{{\left[1+{\left(K_{1}\sqrt{II_{D}}\right)}^{a}\right]}^{\left(\frac{1-\text{n}}{a}\right)}}D$$

where $\overset{\nabla }{\tau }$ is the upper convected time derivative of stress tensor defined as:

(5) $$\overset{\nabla }{\tau }=\frac{\partial \tau }{\partial t}+\left(v\cdot \nabla \right)\tau -L\tau -\tau L^{T}$$

Here *v* is the velocity vector and *L* is the velocity gradient. Considering the steady-state flow and homogeneous (spatially uniform) stress, the time derivative $\frac{\partial \tau }{\partial t}$ and the convective transport term (v⋅∇)τ are equal to zero in Equation (5). The model utilizes four parameters (η_0_, *K*_1_, *a*, *n*) identifiable from shear viscosity and two parameters (*λ*_0_, *K*_2_) identifiable from uniaxial extensional viscosity. It has been shown that if *λ*_0_/*K*_2_ < $\frac{\sqrt{3}}{2}$), the model can be used for a very good description of steady-state uniaxial extensional viscosity for a wide range of real polymer melts [71,75,76,77].

### 3.3. Yao Model

Yao model is a non-Newtonian fluid model with an objective velocity gradient utilizing two different time scales for strain relaxation and rotation recovery, which has been proposed just recently [78]. The model is based on the assumption that firstly, the strain accumulated in the polymer coil is equivalent to the strain in the ideal elastic body for the time equal to relaxation time; secondly, finite extensibility of the chains is handled via “retardation” of unlimited elastic stress growth by introduction of the ceiling stretch *S*_0_; and finally, there is slip during chain disentanglement characterized by the slip velocity gradient D*_S_* and the slip viscosity η*_S_*, which generates slip stress contributing to the total stress. For steady-state flows, it takes the following form:

(6) $$\tau =\frac{\eta_{0}}{\lambda_{0}}B^{*}+2\eta_{S}D_{S}$$

where $B^{*}$ is a modified elastic strain tensor describing the conformation of the molecular coil

(7) $$B^{*}={\left[\exp\left(n_{0}\lambda_{0}\alpha L\right)\cdot \exp\left(n_{0}\lambda_{0}\alpha L^{T}\right)\right]}^{\;\frac{1}{n_{0}}},$$

η*_S_* is the slip viscosity

(8) $$\eta_{S}=\frac{\mu_{S}}{{\left[1+{\left(K_{S}\sqrt{II_{D_{S}}}\right)}^{2}\right]}^{\left(1-\text{k}\right)/2}},$$

and *D_s_* is the portion of deformation rate tensor D contributing to chain slip

(9) $$D_{S}=\left(1-\alpha \right)D_{\;⊥}$$

Here, $II_{D_{S}}$ is the second invariant of deformation rate tensor for the chain slip, $D_{\;⊥}$ represents deformation rate tensor projected onto the principal directions of $B^{*}$, at which chain disentanglement is expected to take place (i.e., along the highest normal stress directions of the polymer coil [78]). α is the function given by the equivalent stretch of the polymer coil defined as

(10) $$\sqrt{1/6\ln B^{*}:B^{*}}=S_{0}\left[\coth\left(3\frac{\sqrt{1/6\ln B:B}}{S_{0}}\right)-\frac{S_{0}}{3\sqrt{1/6\ln B:B}}\right]$$

where *S*_0_ is a ceiling stretch for chain disentanglement, $B^{*}$ is defined by Equation (7) and *B* is an elastic strain tensor defined as

(11) $$B={\left[\exp\left(n_{0}\lambda L\right)\cdot \exp\left(n_{0}\lambda L^{T}\right)\right]}^{\frac{1}{n_{0}}}$$

In order to get $D_{\;⊥}$ appearing in Equation (9), firstly the $B^{*}$ needs to be expressed as $B^{*}=V\cdot \Lambda \cdot V^{-1}$, where Λ is a diagonal tensor; secondly, a rotational tensor *Q* is determined from V by using normalized eigenvectors; thirdly, diagonal components of $Q\cdot D\cdot Q^{\;T\;}$ are extracted (denoted here as *X*_11_, *X*_22_, and *X*_33_); finally, $D_{\;⊥}$ is given by the following equation:

(12) $$D_{\;⊥}=Q^{T}\cdot \left(\begin{array}{ccc} X_{11} & 0 & 0\\ 0 & X_{22} & 0\\ 0 & 0 & X_{33} \end{array}\right)\cdot Q$$

In total, the model utilizes seven adjustable parameters (η_0_, λ_0_, *S*_0_, µ_S_/η_0_, *n*_0_, *k*, *K*_S_).

### 3.4. Extended Yao Model

The recently proposed extended Yao model [79] is simply the original Yao model, wherein, firstly the slip stress is expressed via slip viscosity function η_S_ and slip velocity gradient *D_S_* is neglected (i.e., the term $2\eta_{S}D_{S}=0$); and, secondly, the finite extensibility of individual chains is handled differently—via modified expressions for the relaxation time and modulus by the ceiling stretch *S*_0_ (i.e., by the maximum stretch of a polymer coil) as

(13) $$\lambda =\lambda_{0}\left(1-\frac{S}{S_{0}}\right)\text{ and }G=\frac{G_{0}}{{\left(1-\frac{S}{S_{0}}\right)}^{\beta_{0}}},$$

where *S* is an equivalent stretch defined as $S=\sqrt{\frac{1}{6}\ln B:\ln B}$. This reduces total number of model parameters from seven to five in comparison with the original Yao model. The extended Yao model is then given by the following equations

(14) $$\tau =\frac{\eta_{0}}{\lambda_{0}{\left[1-\frac{S}{S_{0}}\right]}^{\beta_{0}}}B,$$

(15) $$B={\left\{\exp\left[n_{0}\lambda_{0}a_{T}\left(1-\frac{S}{S_{0}}\right)L\right]\cdot \exp\left[n_{0}\lambda_{0}a_{T}\left(1-\frac{S}{S_{0}}\right)L^{T}\right]\right\}}^{\frac{1}{n_{0}}},$$

where *B* is the generalized Finger tensor representing the elastic strain accumulated in the polymer coil. The model utilizes five adjustable parameters (η_0_, λ_0_, *S*_0_, *n*_0_, β_0_). It is important to mention that according to Yao [79], the β_0_ is the strain-hardening parameter ($0\leq \beta_{0}\leq 1$), *n*_0_ is the index of rotational recovery (1≤n≤2) and *S*_0_ is the celling stretch for disentanglement (typically in the range of 1 to 3).

## 4. Results and Discussion

The generalized Maxwell model was employed to fit the measured frequency-dependent loss and storage moduli at reference temperature 180 °C for virgin and degraded PPs to generate discrete relaxation spectra (in terms of finite number of λ_k_ and *G*_k_ pairs), which are provided in Table 1.

Average relaxation time, $\bar{\lambda }$, for each sample was determined according to [80] as follows:

(16) $$\bar{\lambda }=\frac{\sum_{k=1}^{N}G_{k}\lambda_{k}^{2}}{\sum_{k=1}^{N}G_{k}\lambda_{k}}$$

**Figure 1:**
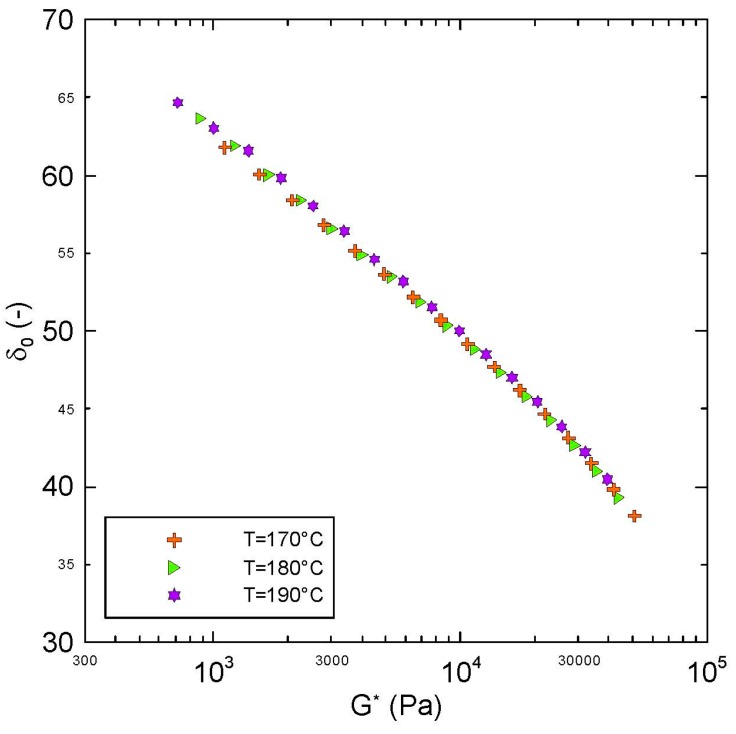
Van Gurp-Palmen plot for virgin polypropylene (PP).

**Figure 2:**
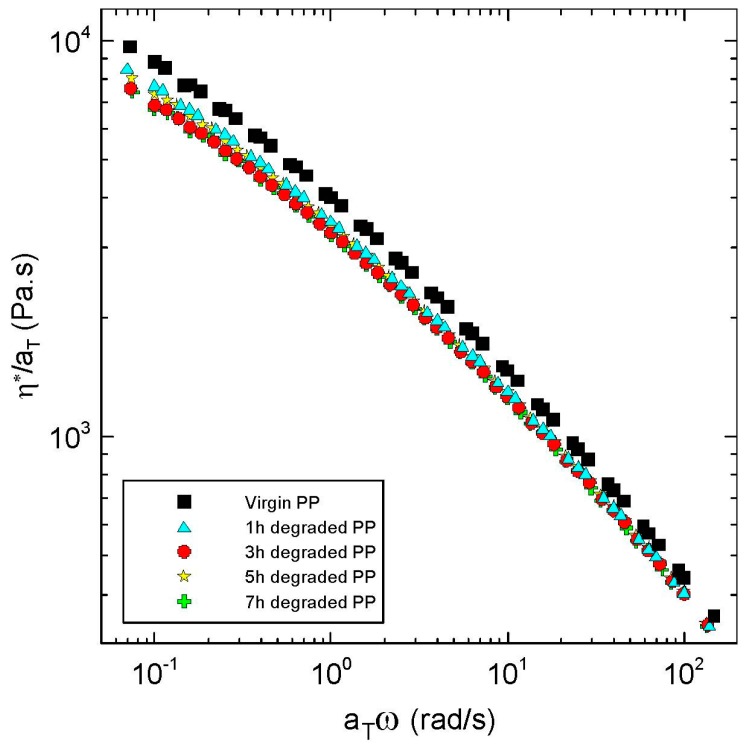
Frequency-dependent complex viscosity, η*, for virgin and degraded polypropylenes at 180 °C.

**Figure 3:**
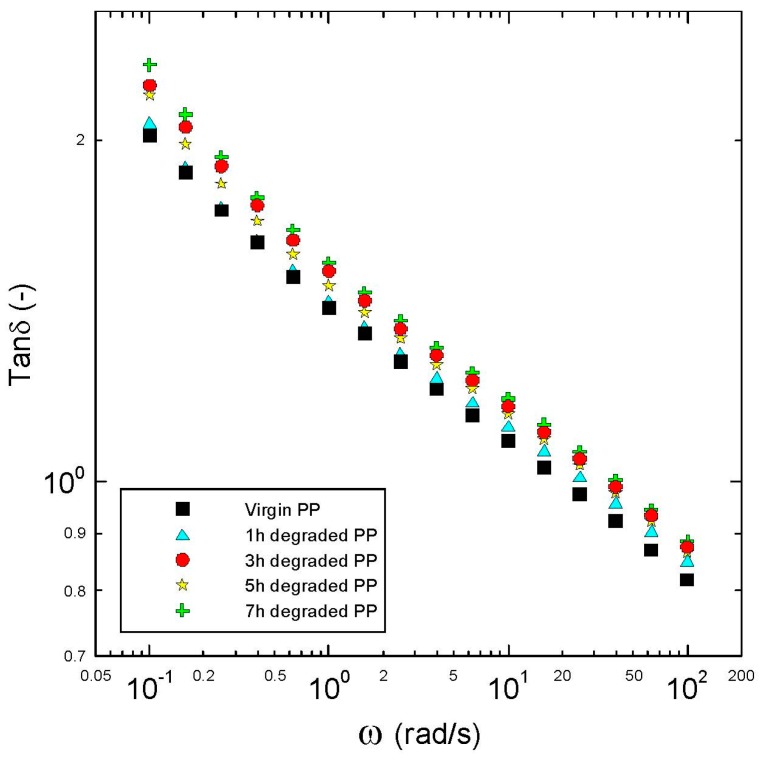
Frequency-dependent Tanδ for virgin and degraded polypropylenes at 180 °C.

**Figure 4:**
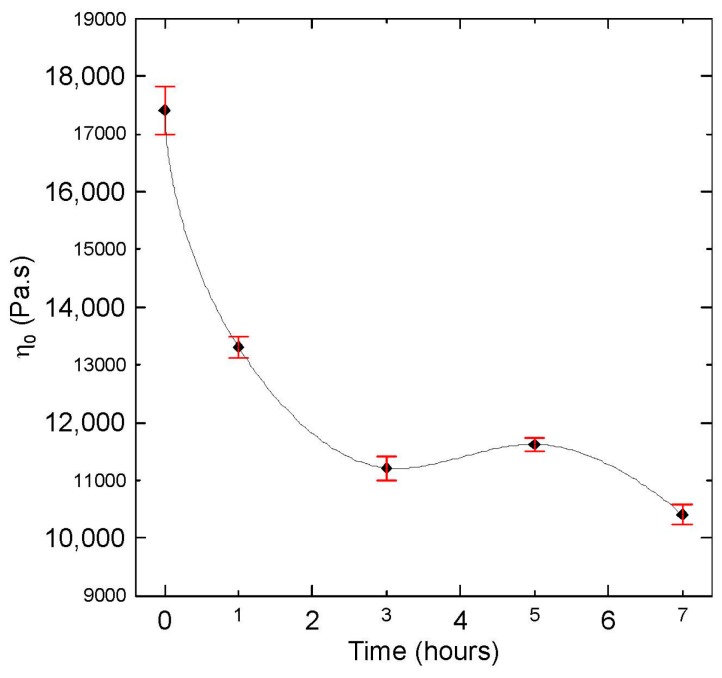
Newtonian viscosity, η_0_, for virgin and degraded polypropylenes obtained from shear creep measurements at 180 °C.

**Figure 5:**
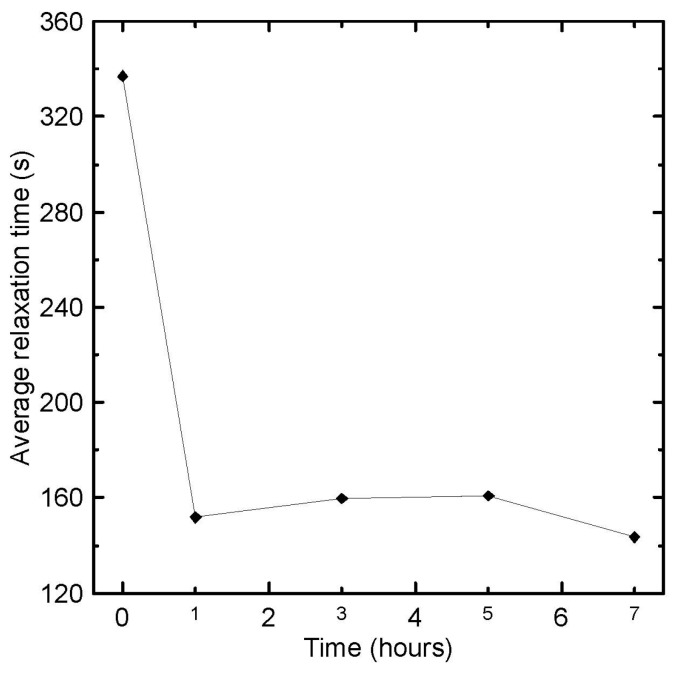
Average relaxation time, calculated via Equation (16) by using discontinues relaxation spectra, plotted as the function of degradation time for investigated polypropylene at 180 °C.

Basic rheological measurements in the small-amplitude oscillatory shear flow together with the average relaxation time are summarized in Figure 1, Figure 2, Figure 3, Figure 4 and Figure 5, from which the following conclusions can be formulated.

Firstly, it can be considered that the tested polypropylene is thermorheologically simple because all measured points coincide into one single curve in the van Gurp-Palmen plot, which is provided in Figure 1. This is typical for the star-like branched polypropylenes but not for the polyethylenes having typically tree-like branching and thermorheologically complex behavior [27]. Thermorheological simplicity of the tested polymer sample justifies utilization of the time-temperature superposition principle to generate master curves in this work.

**Figure 6:**
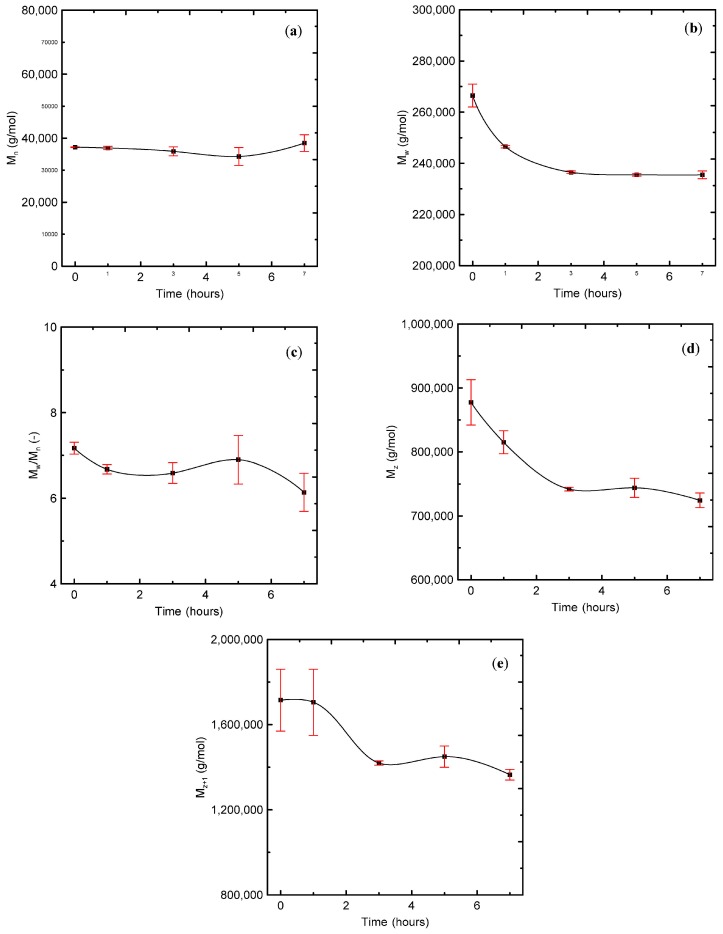
Effect of degradation time on the molecular weight distribution of investigated polypropylene. (**a**) Number average molecular weight (*M*_n_); (**b**) Weight average molecular weight (*M*_w_); (**c**) Polydisperzity index (*M*_w_/*M*_n_); (**d**) *M*_z_ average molecular weight; (**e**) *M_z_*_+1_ average molecular weight.

Secondly, complex viscosity (Figure 2), shear elasticity captured through Tanδ here (Figure 3), Newtonian viscosity (Figure 4) and average relaxation time (Figure 5) firstly decrease with the degradation time, then surprisingly increase up to the local maximum at five hours and finally decrease again. This indicates simultaneous occurrence of the chain scission and recombination reactions during thermal degradation of the tested polypropylene. This rheological observation is in very good correspondence with the polydispersity index *M*_w_/*M*_n_, *M*_z_ and *M*_z+1_ molecular weight averages that show exactly the same non-monotonic behaviour during thermal degradation as shown in Figure 6.

**Figure 7:**
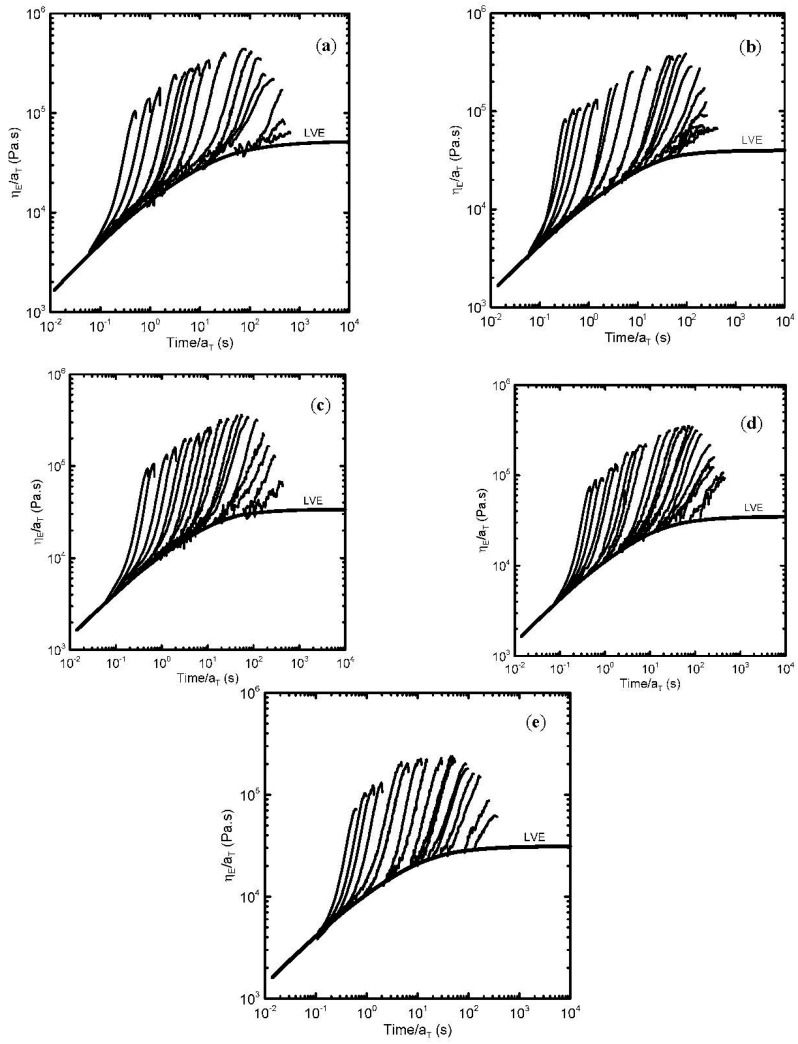
Transient extensional rheology of virgin as well as degraded branched polypropylene obtained from Sentmanat extensional rheometer at different extensional strain rates for 180 °C. (**a**) Virgin PP; (**b**) 1 h degraded PP; (**c**) 3 h degraded PP; (**d**) 5 h degraded PP; (**e**) 7 h degraded PP.

**Figure 8:**
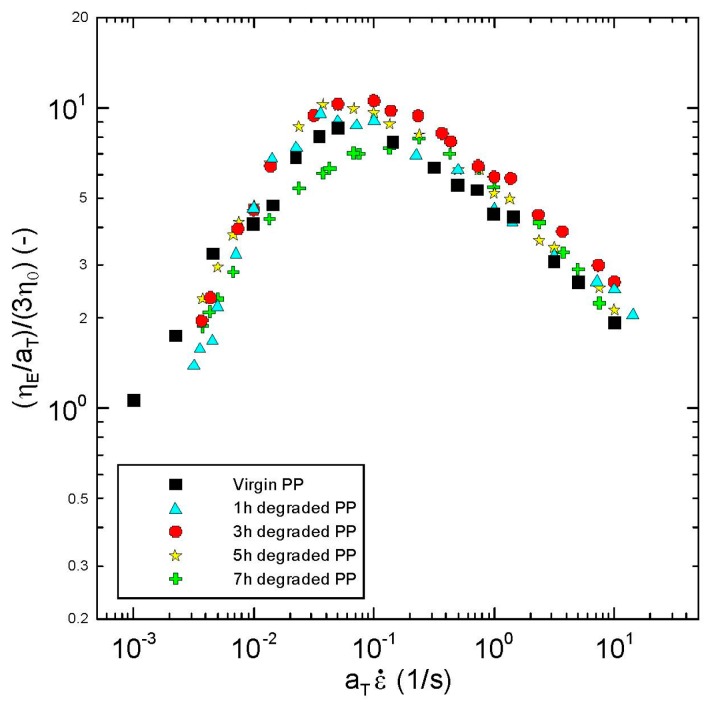
Steady-state extensional viscosity data at 180 °C normalized by the three-fold Newtonian viscosity for virgin and thermally degraded branched polypropylenes.

Transient extensional viscosity data for virgin as well as thermally degraded branched PPs evaluated at different extensional strain rates (in the range 0.001–14.3 s^−1^) are provided in Figure 7. Here LVE represents Linear Viscoelastic Envelope determined from discontinuous relaxation spectrum. The “steady-state” uniaxial extensional viscosity data were determined from the peaks appearing on the transient viscosity curves for corresponding extensional strain rates. Obtained “steady-state” extensional viscosity data, normalized by the 3 times Newtonian viscosity (measure of the extensional strain hardening and chain branching [27,81]), are provided in Figure 8 as the function of the extensional strain rate. Interestingly, the level of the branching firstly increases within first three hours of thermal degradation and then decreases as deduced from the measure of the extensional strain hardening provided in Figure 8. This indicates presence of the recombination reactions between polypropylene chains enhancing the branching level, especially within the first 3 h of thermal degradation.

The complex flow behavior observed in the shear and extensional flows as well as GPC data suggests that during the thermal degradation of branched polypropylene melt, the scission of the longest and branched chains is more dominant than recombination reactions, which leads to production of short branched chains from which some of them recombines together and creates medium length chains with enhanced branching level. Continuous reduction of the longest chains in the polymer melt can explain reduction in Newtonian viscosity and shear elasticity whereas increased amount of medium length chains with enhanced level of branching can explain increase in extensional strain hardening at low degradation times. At higher degradation times, it seems that the process is repeated i.e., the chain scission starts to be more dominant than recombination reactions for medium length chains. Consequent reduction of middle length chains and presence of high amount of short chains with enhanced branching level can explain reduction of extensional strain hardening at long degradation times. This hypothesis is supported by the shift of the maximum steady-state uniaxial extensional viscosity to the higher extensional strain rates range for increased degradation time, as visible in Figure 8.

In the next step, considering validity of the Cox-Merz rule, deformation rate-dependent shear and extensional viscosities were fitted by the Generalized Newtonian law (Equations (1)–(3)) modified White Metzner model (Equations (4)–(5)), Yao model (Equations (6)–(12)) and Extended Yao model (Equations (13)–(15)). All model parameters are summarized in Table 2, Table 3, Table 4 and Table 5. Fitting error for each model was evaluated via the Average Root Mean Squared Error (ARMSE) defined as

(17) $$ARMSE=\frac{1}{2}\left\{\sqrt{\frac{1}{\delta_{S}}\sum_{i=1}^{\delta_{S}}{\left[\text{Log}(\eta_{\text{S},\text{i}})-\text{Log}({\hat{\eta }}_{\text{S},\text{i}})\right]}^{\;2}}+\sqrt{\frac{1}{\delta_{E}}\sum_{i=1}^{\delta_{E}}{\left[\text{Log}(\eta_{\text{E},\text{i}})-\text{Log}({\hat{\eta }}_{\text{E},\text{i}})\right]}^{\;2}}\right\}$$

where δ is the number of measured points, $\eta_{i}$ and ${\hat{\eta }}_{i}$ represent measured and predicted points and indexes *S* and *E* stand for Shear and Extensional viscosities (see Table 6).

**Figure 9:**
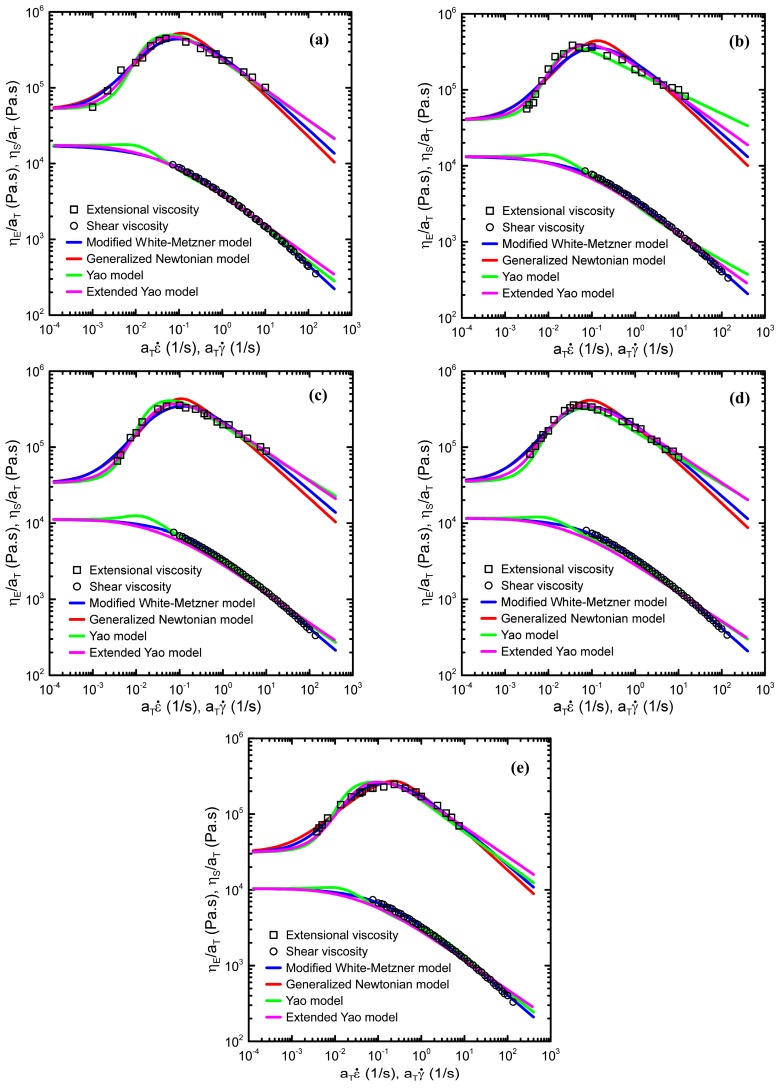
Comparison between the measured steady-state shear and uniaxial extensional viscosities and model fits at 180 °C. (**a**) Virgin PP; (**b**) 1 h degraded PP; (**c**) 3 h degraded PP; (**d**) 5 h degraded PP; (**e**) 7 h degraded PP.

**Figure 10:**
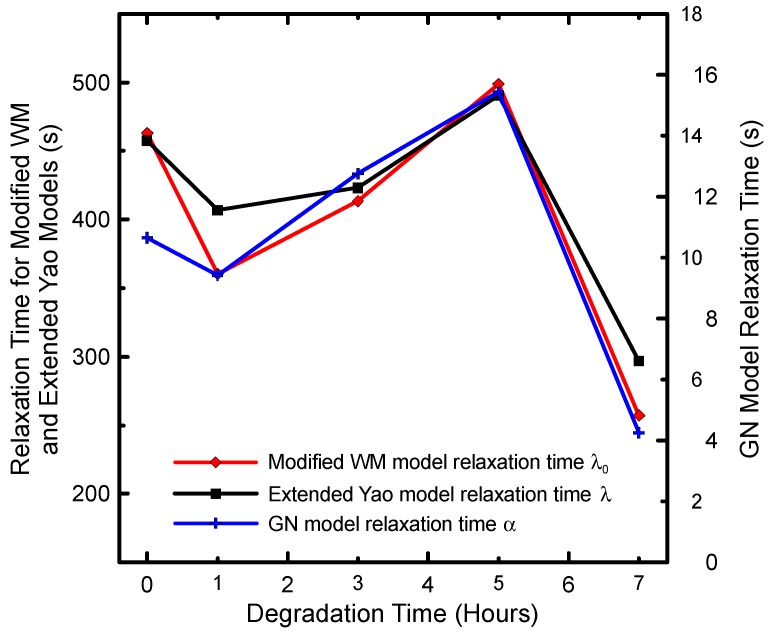
Model relaxation times for branched PP at 180 °C for different degradation times (raw data are provided in Table 2, Table 3 and Table 5).

Comparison between model fitting lines and the measured data are provided in Figure 9. As can be seen, all four models have the capability to describe the measured data very well. Detailed analysis of model parameters has revealed that, firstly, the model macroscopic relaxation time plotted as the function of degradation time (see Figure 10) follows the same non-monotonic trend with local maximum at 5 hours, which was observed experimentally in basic shear flow characteristics as well as in the *M*_z_ and *M*_z+1_ molecular weight averages, as discussed above. This suggests that during the thermal degradation of branched PP, the size and weight of the characteristic polymer coil follows the same non-monotonic trend.

**Figure 11:**
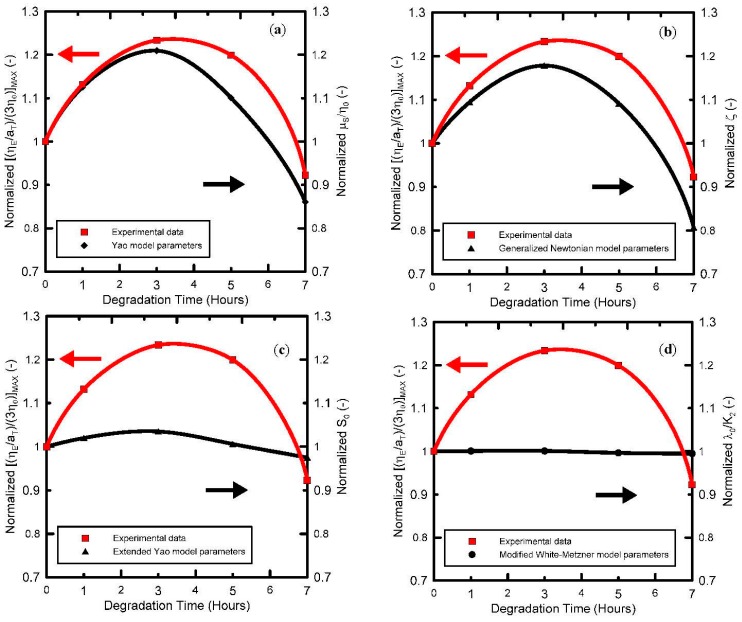
Correlation between normalized uniaxial extensional viscosity and normalized model parameters for branched PP at 180 °C for different degradation times. (**a**) Yao model; (**b**) Generalized Newtonian model; (**c**) Extended Yao model; (**d**) Modified White-Metzner model.

Secondly, it has been found that the strain-hardening parameter ζ in the Generalized Newtonian model, strain-hardening *λ*_0_/*K*_2_ parameter in the modified White Metzner model as well as μ_S_/η_0_ parameter in the Yao model and the ceiling stretch for disentanglement *S*_0_ in the extended Yao model firstly increases with the degradation time, reaching the local maximum at 3 h of thermal degradation, and then decreases, i.e., all the parameters follow a non-monotonic trend in the extensional strain-hardening level with respect to degradation time as observed experimentally. This suggests that all four models can be used to quantify the level of the branching for polypropylenes via their parameters (ζ, *λ*_0_/*K*_2_, μ_S_/η_0_ and *S*_0_) in a very simple way due to availability of analytical solutions for extensional viscosities and analytical (or in the case of the Extended Yao model, nearly analytical) solutions for steady-state shear viscosity, which makes the experimental data a fitting procedure, both simple and straightforward. It is interesting to note that Yao and Generalized Newtonian models are able to quantify level of extensional strain hardening (i.e., the maximum steady-state uniaxial extensional viscosity divided by three-fold Newtonian viscosity) as a function of degradation time via their parameters, not only quantitatively but also qualitatively as shown in Figure 11. It is important to mention that Yao model parameter μ_S_/η_0_ represents the relative resistance against the slip of polymer coils, which depends on their overlap as well as on their capability to create entanglements during the flow. As it has already been shown, the size and the weight of polymer coils during thermal degradation decrease during the first 3 h. Thus, the observed increase in the Yao parameter μ_S_/η_0_ during the first 3 h of thermal degradation can be explained via enhanced capability to create entanglements between the coils due to increased number of branches but having reduced molecular weight in comparison with the virgin polymer. On the other hand, decrease in μ_S_/η_0_ for degradation lasting more than 3 h can be interpreted as a decrease in number of branches.

**Figure 12:**
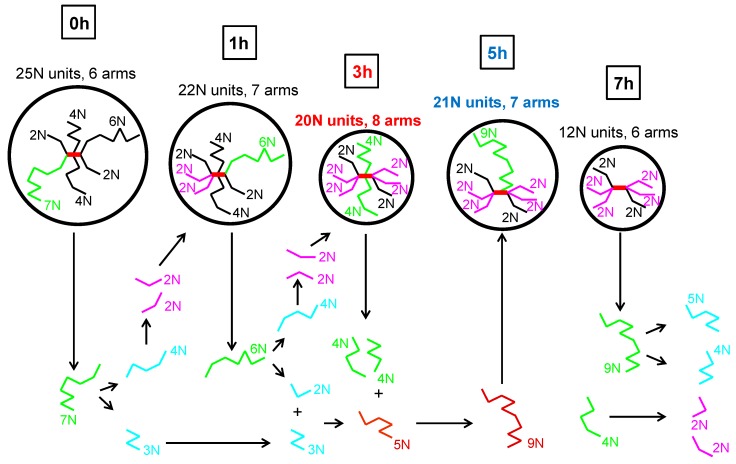
Simplified visualization of possible changes in “characteristic” polymer coil for investigated branched PP at 180 °C during thermal degradation.

Therefore, local increase in shear viscosity, elasticity, relaxation time and molecular weight of the coil at 5 h of thermal degradation could be explained by the presence of a lower number of branches where some of them have higher molecular weight in comparison with the 3 h degraded sample. Simplified visualization of possible changes in “characteristic” star polymer coil during thermal degradation according to collected experimental data and the above-described hypothesis is provided in Figure 12. Here, the coil size is proportional to the molecular weight, the longest branches are represented by the green colour, and branches formed via branch scission and recombination reactions are represented here by blue/purple and brown colours, respectively. It should also be mentioned that the presented image of molecular changes during thermal degradation of branched polypropylene is consistent with the Bueche degradation theory and conclusions from [56], i.e., that the longest chains break first.

## 5. Conclusions

In this work, thermal stability of the branched polypropylene was investigated via shear and extensional rheology, Gel Permeation Chromatography (GPC) measurements and constitutive equations. It was shown that the studied polypropylene is thermorheologically simple showing simultaneous occurrence of chain scission and branching during thermal degradation. In more detail, complex viscosity, shear elasticity, Newtonian viscosity and average relaxation time was found to decrease with the degradation time within first 3 h, then increase up to the local maximum at five hours and finally decrease again indicating simultaneous occurrence of the chain scission and recombination reactions. This rheological observation was found to be in good correspondence with the changes in polydispersity index *M*_w_/*M*_n_, *M*_z_ and *M*_z+1_ molecular weight averages. Consequent analysis of the virgin and thermally degraded polymer melts in the uniaxial extensional flows has revealed that the level of extensional strain hardening (i.e., the level of chain branching) increases within the first three hours of thermal degradation and then decreases. This indicated dominance of recombination and scission reactions on the chain branches before and after 3 h of thermal degradation, respectively.

It has been found that the utilized Generalized Newtonian law, modified White Metzner model, Yao model and Extended Yao model have the capability to describe the measured steady state shear and extensional rheology of the virgin as well as degraded branched polypropylenes. Analysis of the rheological data via the utilized model parameters has revealed that, firstly, the model macroscopic relaxation time plotted as the function of degradation time has been found to follow the same non-monotonic trend with local maximum at 5 h, which was observed experimentally in basic shear flow and molecular weight characteristics (*M*_w_/*M*_n_, *M*_z_, *M*_z+1_); Secondly, it has been found that the strain hardening parameter *ζ* in the Generalized Newtonian model, strain hardening *λ*_0_/*K*_2_ parameter in the modified White Metzner model as well as μ_S_/η_0_ parameter in the Yao model and the ceiling stretch for disentanglement *S*_0_ in the extended Yao model follows the non-monotonic trend in the extensional strain hardening level with respect to degradation time as observed experimentally, i.e., all four models were found to have the capability to quantify the level of the branching for virgin as well as degraded polypropylenes. Interestingly, Yao and Generalized Newtonian models showed the capability to quantify level of extensional strain hardening (i.e., the maximum steady-state uniaxial extensional viscosity divided by three-fold Newtonian viscosity) as a function of degradation time not only quantitatively but also qualitatively. Finally, based on the Yao’s μ_S_/η_0_ parameter (characterizing the resistance against the slip of the coil), it was suggested that the local maximum at 5 h in shear rheological characteristics and molecular weight could be explained by the presence of high molecular weight branches. On the other hand, the local minimum in shear rheology characteristics and molecular weights with simultaneous occurrence of maximum in extensional strain hardening observed at 3 h of thermal degradation was suggested to be due to the presence of a high number of short branches.

## Figures and Tables

**Table 1:** Maxwell model parameters for all tested polymer samples at *T* = 180 °C.

	Virgin PP		1 h Degraded PP		3 h Degraded PP		5 h Degraded PP		7 h Degraded PP	
i	λ_i_ (s)	*G*_i_ (Pa)	λ_i_ (s)	*G*_i_ (Pa)	λ_i_ (s)	*G*_i_ (Pa)	λ_i_ (s)	*G*_i_ (Pa)	λ_i_ (s)	*G*_i_ (Pa)
1	0.00677	52,148.6	0.00715	48,451.8	0.00732	48,414.1	0.00749	47,962.5	0.00744	47,463.2
2	0.03283	12,150.5	0.03446	10,454.9	0.03517	9,926.72	0.03591	9,950.26	0.03567	9,680.62
3	0.15912	7,033.90	0.16603	5,974.36	0.16899	5,688.47	0.17213	5,821.17	0.17111	5,616.08
4	0.77125	2,521.01	0.79986	2,122.36	0.81207	1,914.91	0.82499	1,937.14	0.82079	1,848.84
5	3.73822	898.698	3.85341	761.059	3.90237	686.725	3.95403	780.312	3.93727	712.969
6	18.1189	264.527	18.5642	252.45	18.7526	189.403	18.951	160.582	18.8867	144.403
7	87.8211	31.1191	89.4349	15.7774	90.1149	11.2843	90.8289	14.2091	90.5977	11.1562
8	425.663	3.85780	430.862	1.68866	433.043	1.39873	435.327	1.44066	434.588	1.16212
9	2,063.16	0.28283	2,075.72	0.02515	2,080.97	0.02156	2,086.45	0.06774	2,084.68	0.04436
10	10,000	0.03582	10,000	0.01378	10,000	0.01270	10,000	0.01111	10,000	0.00926

**Table 2:** Generalized Newtonian model parameters for *T* = 180 °C, ψ = 8.

Sample Name	η_0_ (Pa.s)	*K*_1_ (s)	*a* (-)	*n* (-)	α (s)	β (-)	ζ (-)	*A* (Pa.s)
Virgin PP	17,413.56	9.462	0.5252	0.4718	10.64815	0.011488	0.015880	1.86 × 10^−16^
1 h Degraded PP	13,307.62	8.301	0.5759	0.4870	9.41753	0.016025	0.017381	2.00 × 10^−16^
3 h Degraded PP	11,215.75	8.691	0.6462	0.5142	12.75510	0.022095	0.018715	2.04 × 10^−16^
5 h Degraded PP	11,620.91	7.365	0.6267	0.4974	15.43329	0.019541	0.017331	2.08 × 10^−16^
7 h Degraded PP	10,408.44	7.350	0.6651	0.5108	4.256025	0.004785	0.012814	2.06 × 10^−16^

**Table 3:** Modified White-Metzner model parameters for *T* = 180 °C.

Sample Name	η_0_ (Pa.s)	*K*_1_ (s)	*a* (-)	*n* (-)	λ_0_(s)	λ_0_/*K*_2_
Virgin PP	17,413.56	9.462	0.5252	0.4718	462.91	0.844665
1 h Degraded PP	13,307.62	8.301	0.5759	0.4870	360.32	0.845266
3 h Degraded PP	11,215.75	8.691	0.6462	0.5142	413.35	0.845383
5 h Degraded PP	11,620.91	7.365	0.6267	0.4974	498.83	0.841680
7 h Degraded PP	10,408.44	7.350	0.6651	0.5108	257.23	0.840236

**Table 4:** Yao model parameters for *T* = 180 °C.

Sample Name	η_0_ (Pa.s)	λ_0_ (s)	*S*_0_ (-)	*µ*_s_/η_0_ (-)	*n*_0_ (-)	*K*_S_ (s)	*k* (-)
Virgin PP	17,413.60	150.933	2.159	9.955	1.668	5.102	0.611
1 h Degraded PP	13,307.6	133.713	1.990	11.204	1.741	22.002	0.731
3 h Degraded PP	11,215.75	158.497	2.476	12.041	1.447	5.509	0.650
5 h Degraded PP	11,620.9	131.419	1.582	10.958	2.141	7.607	0.658
7 h Degraded PP	10,408.44	129.008	2.115	8.567	1.563	2.344	0.584

**Table 5:** Extended Yao model parameters for *T* = 180 °C.

Sample Name	η_0_ (Pa.s)	*λ* (s)	*S*_0_ (-)	*β* (-)	*n* (-)
Virgin PP	17,413.60	457.556	2.8812	0.592302	1
1 h Degraded PP	13,307.6	406.645	2.93809	0.59207	1
3 h Degraded PP	11,215.75	423.1135	2.981601	0.612026	1
5 h Degraded PP	11,620.9	490.3696	2.898281	0.624356	1
7 h Degraded PP	10,408.44	296.6814	2.810061	0.609387	1

**Table 6:** Fitting error (average root mean squared error—ARMSE) for each utilized model.

Model Name	ARMSE for virgin PP	ARMSE for 1 h degraded PP	ARMSE for 3 h degraded PP	ARMSE for 5 h degraded PP	ARMSE for 7 h degraded PP	ARMSE for for all tested samples
Generalized Newtonian model	0.038075361	0.057256216	0.037801435	0.031291613	0.02292228	0.037469381
Modified White-Metzner model	0.027314726	0.050005197	0.027266301	0.024427789	0.016706735	0.029144150
Yao model	0.051247568	0.052358684	0.033786887	0.051634911	0.061832914	0.050172193
Extended Yao model	0.035187159	0.037493283	0.028874476	0.033933070	0.039698414	0.035037281
